# Prognostic value of left atrial mechanics in cardiac light-chain amyloidosis with preserved ejection fraction: a cohort study

**DOI:** 10.1186/s12872-022-02589-7

**Published:** 2022-04-15

**Authors:** Xiao-hang Liu, Jia-yu Shi, Ding-ding Zhang, Fu-wei Jia, Xue Lin, Yan-lin Zhu, Jun-ling Zhuang, Li-gang Fang, Wei Chen

**Affiliations:** 1 Department of Cardiology, Peking Union Medical College Hospital, Chinese Academy of Medical Sciences and Peking Union Medical College, Beijing, 100730 China; 2 Department of Cardiology, Affiliated Hospital of Nantong University, Medical School of Nantong University, Jiangsu, China; 3 Medical Research Center, Peking Union Medical College Hospital, Chinese Academy of Medical Sciences and Peking Union Medical College, Beijing, China; 4 Department of Hematology, Peking Union Medical College Hospital, Chinese Academy of Medical Sciences and Peking Union Medical College, Beijing, China

**Keywords:** Atrial strain, Amyloid, Speckle tracking echocardiography, Prognosis

## Abstract

**Background:**

Light-chain amyloidosis is a plasma cell disorder associated with poor outcomes, especially when the heart is involved. The characteristics of left atrial (LA) function and its prognostic implications in cardiac amyloidosis (CA) have not been fully investigated.

**Methods:**

Between April 2014 and June 2019, 93 patients with a diagnosis of CA, normal left ventricular ejection fraction (LVEF) and sinus rhythm were included. Their clinical, baseline echocardiographic and follow-up data were investigated. LA function, including LA strain and strain rate, was assessed using 2D speckle tracking echocardiography in different LA functional phases.

**Results:**

Among all patients, 38 (40.9%) died. Multivariate Cox regression analyses showed that LA mechanics regarding LA reservoir and booster pump functions were independent predictors for overall survival. Traditional echocardiographic parameters for LA structure like LA volume index and LA width were not associated with mortality. Moreover, LA strain and strain rate in reservoir and contractile phases improved the discrimination and goodness of fit of the conventional prognostic model, the Mayo criteria 2004 and 2012, in our study population. Decreased LA mechanics were associated with impaired left ventricular (LV) systolic and diastolic function, and LA reservoir and contractile functions were associated with LA structure.

**Conclusions:**

Assessment of LA reservoir and contractile functions via 2D speckle tracking echocardiographic LA mechanical indices provide clinical and prognostic insights into cardiac light-chain amyloidosis patients, especially those with preserved EF and sinus rhythm. Emphasizing the monitoring of LA function may be beneficial for the prognosis prediction of CA.

**Supplementary Information:**

The online version contains supplementary material available at 10.1186/s12872-022-02589-7.

## Background

The amyloidoses are a group of protein-folding disorders characterized by the infiltration of proteinaceous deposits called amyloid in different organs, including the heart [1]. Although transthyretin cardiac amyloidosis (ATTR-CA) is increasingly being diagnosed and is likely the most common type of cardiac amyloidosis, systemic light-chain (AL) amyloidosis is still another one of the 2 commonest forms of amyloidosis [2]. Cardiac amyloidosis (CA) carries the worst prognosis among all organs involved. If untreated, amyloid involvement of the heart is associated with a median survival time of 6 months for patients with light-chain amyloidosis [3]. Therefore, the early recognition of CA is critical.

Among the various methods used for evaluating cardiac morphology and function, transthoracic echocardiography, a convenient noninvasive method, is widely used for the initial detection and is included in the definition of CA in clinical practice. Cardiac involvement in AL amyloidosis is confirmed if either a histologically positive result is found in endomyocardial biopsy (EMB) or if a mean wall thickness > 12 mm without other cardiac causes is detected by echocardiography in a patient with a positive biopsy result at an alternate site [4]. Other typical echocardiographic features include left atrial (LA) enlargement, left ventricular (LV) diastolic dysfunction and, at the late stage, a restrictive cardiac pattern [5].

LA enlargement, as a typical echocardiographic finding, is common in CA [6]. Not only LV systolic and diastolic dysfunction but also intrinsic LA failure due to amyloid infiltration contribute to LA involvement [7]. In recent years, it has been shown that LA size could be an independent predictor of cardiovascular events and mortality in CA patients [5, 8]. However, its prognostic value in the specific patient group at less severe disease stage without risk factors such as impaired ejection fraction and atrial fibrillation has not been evaluated. Meanwhile, LA remodeling does not necessarily reflect LA function, although it is often accompanied by a change in LA performance. More recently, LA strain and strain rate obtained by 2D speckle tracking have been demonstrated to be feasible and reproducible in evaluating LA function in the reservoir, conduit, and booster pump phases [9]. The quantitative characterization of LA mechanics during all LA functional phases in CA patients has been investigated and compared with those in normal controls [7]. However, little data exists on the prognostic utility of LA mechanics in CA.

The aim of the study was to investigate the prognostic value of LA mechanics in cardiac light-chain amyloidosis patients with preserved ejection fraction (EF) that were not at the end stage of CA with significant systolic dysfunction and without atrial fibrillation which was a marker of significant damage of atrium. In this study population, we would demonstrate whether LA mechanics could be used as a predictor for all-cause mortality, add incremental prognostic value to conventional clinical (Mayo stage 2004 and 2012) and echocardiographic predictors, above and beyond LA size. Therefore, we sought to analyze the clinical, echocardiographic and follow-up data of cardiac light-chain amyloidosis patients.

## Methods

### Study population

We screened patients diagnosed with cardiac amyloidosis at Peking Union Medical College Hospital between April 2014 and June 2019. All patients had a histological demonstration of amyloid deposits and confirmation of the fibril type. Cardiac involvement was identified after fulfilling the following criteria: LV hypertrophy (> 12 mm) in the absence of other causes existing together with positive pathological results from EMB or other involved organs. If EMB revealed the amorphous deposits of amyloid fibrils, a thickened LV wall was not necessary [10]. The exclusion criteria were a nonsinus rhythm, uncontrolled blood pressure, a history of ischemic heart disease, other etiologies of amyloidosis and baseline echocardiographic LVEF < 50%. This study was approved by the ethics committee of Peking Union Medical College Hospital and complied with the Helsinki Declaration (as revised in 2013).

### Echocardiography

All subjects underwent comprehensive 2D echocardiography with conventional Doppler and tissue Doppler imaging on a Vivid E9 (GE Medical Systems, Milwaukee, Wisconsin). All images were obtained at a frame rate of 50 to 70 fps. The analysis was performed offline on commercially available software (Echopac, GE Medical Systems, Milwaukee, Wisconsin) by a investigator blinded to the clinical information. All measurements and analyses of standard echographic and Doppler parameters were conducted according to current recommendations [11–13]. Images with unacceptable quality were excluded.

**Fig. 1:**
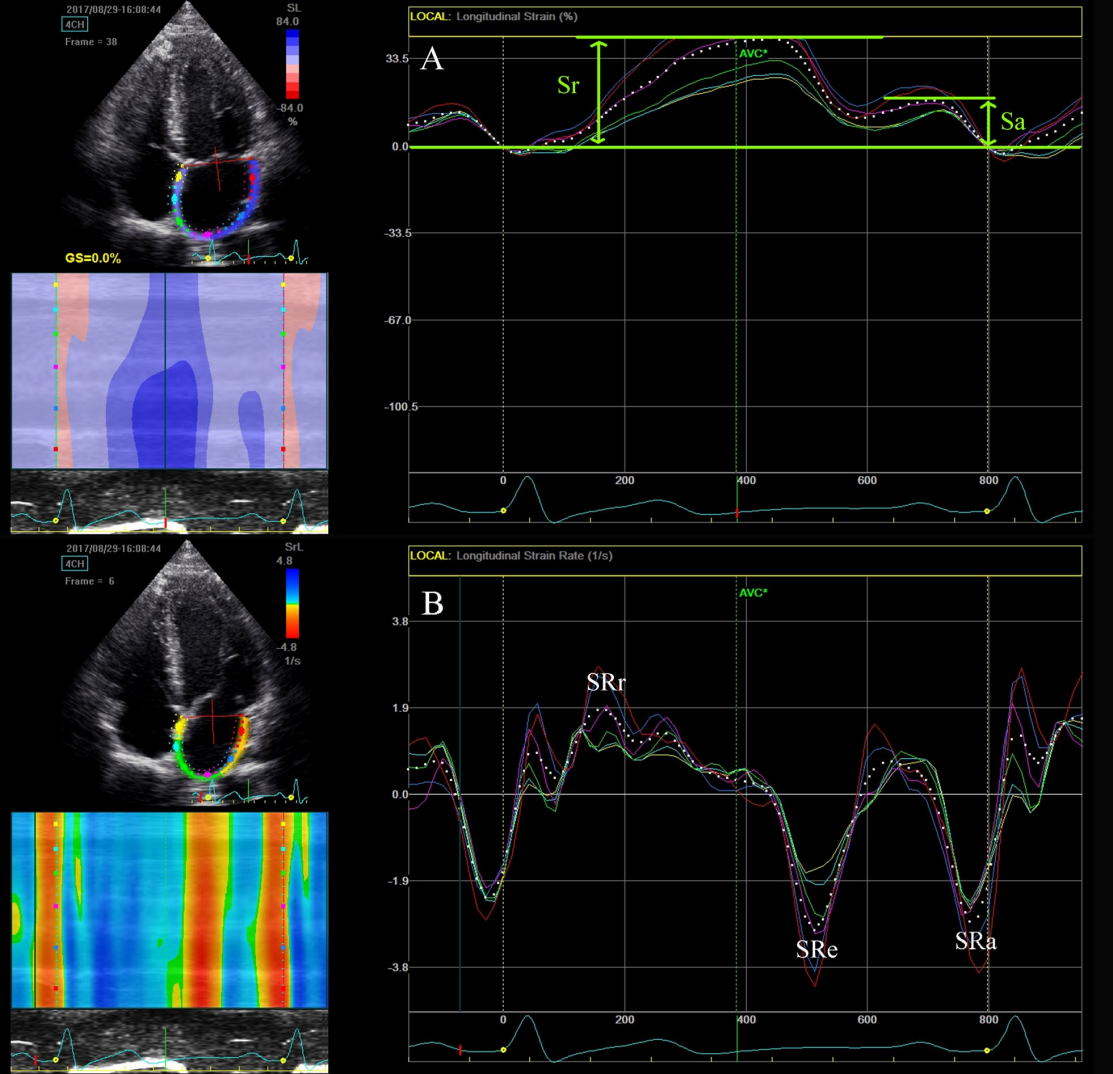
Speckle-tracking echocardiographic left atrial strain waves. **A** Left atrial longitudinal strain waves. Sr, LA total strain; Sa, LA active strain. **B** Left atrial longitudinal strain rate waves. SRr, LA total strain rate; SRe, LA passive strain rate; SRa, LA active strain rate

To date, no dedicated software or guidelines for LA strain have been released. The components of LA strain in our study were defined as follows: LA total strain = peak longitudinal LA strain; LA active strain = longitudinal LA strain measured between the onset of the P wave and the onset of the QRS complex; LA passive strain = LA total strain-LA active strain (Fig. 1) (surrogates of the LA reservoir, contractile and conduit function, respectively). The software designed for LV analysis was used to study LA strain. The LA endocardial border was manually traced in apical 4- and 2-chamber views and subsequently adjusted according to the actual thickness of the LA wall. LA longitudinal deformation was quantified and averaged for the 6 segments divided by the software. LA stiffness index was calculated as the ratio of E/e’ to LA total strain as previously defined [14]. To evaluate the interobserver and intraobserver reproducibility of LA strain and strain rate, data from 10 randomly selected patients were analyzed by the same observer within 2 weeks and by a second independent observer.

In addition to LA strain, LV global longitudinal strain (GLS) was also calculated as the average LV longitudinal strain across the 18 segments obtained via apical 4-, 3- and 2-chamber views as previously described [15].

### Follow-up

Patients were evaluated after each cycle of chemotherapy. For patients who had not visited the clinic for 6 months, vital status was updated via telephone at 3-month intervals. Follow-up was completed in May 2020. The primary endpoint was overall survival (OS). Hematologic response was assessed according to the 2012 International Society of Amyloidosis (ISA) criteria [16].

### Statistical analysis

Categorical variables are displayed as numbers (percentage), and continuous variables are expressed as the mean ± standard deviation (SD) or median [interquartile range (IQR)] based on normal or nonnormal distribution. Intergroup differences were assessed by Student’s t-test for quantitative variables or Mann–Whitney U test as appropriate, and chi-square test for categorical variables. Univariate Cox regression analyses were used to determine the association between clinical and echocardiographic parameters and survival. Prognostic value of LA mechanics was further examined via multivariate Cox analysis with forward stepwise regression performed in two models with entry and retention at a significance level of 0.05. Model 1 was adjusted for the Meta-Analysis Global Group in Chronic Heart Failure (MAGGIC) risk score (including age, ejection fraction, creatinine, diabetes mellitus, chronic obstructive pulmonary disease, systolic blood pressure, body mass index, heart rate, current smoker, disease duration, New York Heart Association class, beta blocker use and angiotensin-converting enzyme inhibitor use) [17] and Mayo stage 2004. Model 2 was adjusted for the model 1 variables + statistically significant echocardiographic indices in the univariate Cox regression analysis. Receiver operating characteristic (ROC) curves were constructed to determine the optimal cut-off value for the variables retained in the Cox multivariate regression analyses. Overall survival was assessed by Kaplan–Meier analyses, with comparisons performed by the log-rank test. Pearson’s correlation coefficient was used to evaluate the correlation between LA mechanical indices and other clinical and echocardiographic findings. These analyses were performed using SPSS version 22 (IBM, Armonk, NY, USA).

To determine the incremental prognostic utility of LA mechanics beyond Mayo stage 2004 and 2012, we used a combination of tests with R software (version 3.6) using survival (version 3.1-12) and survIDINRI (version 1.1-1) packages, including the Harrell C-statistic, integrated discrimination improvement (IDI) and net reclassification improvement (NRI) for discrimination; and the likelihood ratio test and Bayes information criteria (BIC) for goodness of fit. The prognostic value of LA mechanics + Mayo stage 2004 or 2012 was compared with that of Mayo stage 2004 or 2012 alone.

A *P* value < 0.05 (two-sided) was considered statistically significant.

## Results

### Demographics and clinical data

A total of 155 patients were initially included, 51 of them were excluded as the following reasons: 16 with arrhythmia (12 atrial fibrillation), 7 with coronary artery disease, 5 with transthyretin amyloidosis, 2 with uncontrolled blood pressure and 21 with baseline echocardiographic LVEF < 50%. An additional 11 patients were excluded because of unacceptable image quality for speckle tracking strain analysis. For the final analyses, 93 patients were available.

Males and females accounted for a similar proportion (53.8% versus 46.2%), and the average age at diagnosis was 58.6 ± 9.4 years. Mayo criteria 2004 stages I, II and III accounted for 11.8%, 38.7% and 49.5%, respectively. During a median follow-up period of 21.0 (IQR 7–48) months, 38 (40.9%) patients reached the primary endpoint after 11.3 ± 13.1 months. Table 1 displayed the comparison of baseline characteristics between survivors and non-survivors.

**Table 1:** Comparisons of demographic and clinical characteristics between survivors and non-survivors

Variables	Total (n = 93)	Survivors (n = 55)	Non-survivors (n = 38)	*P* value
Age, years	58.6 ± 9.4	58.1 ± 9.3	59.3 ± 9.7	0.563
Male sex, n (%)	50 (53.8%)	26 (47.2%)	24 (63.2%)	0.131
Systolic blood pressure, mmHg	105.3 ± 16.2	106.4 ± 15.3	103.7 ± 17.6	0.468
Diastolic blood pressure, mmHg	68.5 ± 10.4	68.8 ± 10.6	68.1 ± 10.3	0.784
Heart rate, bpm	82.0 ± 13.6	80.7 ± 13.9	83.8 ± 13.3	0.288
BMI, kg/m^2^	22.3 ± 3.0	21.8 ± 2.8	23.0 ± 3.2	0.077
Smoking, n (%)	34 (36.6%)	18 (32.7%)	16 (42.1%)	0.356
eGFR, ml/min/1.73 m^2^	97.2 ± 32.6	98.6 ± 28.8	95.3 ± 37.9	0.661
NYHA class 3 or 4, n (%)	36 (38.7%)	13 (23.6%)	23 (60.5%)	0.001
NT-proBNP, median [IQR]	2514 [818–7545]	2449 [855–4615]	6261 [2112–8912]	0.006
Intact M protein, n (%)	37 (39.8%)	26 (47.3%)	11 (28.9%)	0.076
Intact M concentration, g/L, median [IQR]	4.7 [2.0–9.1]	3.7 [1.8–7.1]	6.9 [3.6–9.3]	0.454
Bone marrow plasma cells, %, median [IQR]	4.0 [2.3–10.0]	3.5 [2.5–9.6]	5.0 [2.0–10.5]	0.826
dFLC, mg/L, median [IQR]	216.2 [90–387]	198.5 [40.8–382]	241.5 [171.5–447]	0.535
Involved light chain type λ, n (%)	78 (83.9%)	45 (81.8%)	33 (86.8%)	0.517
Mayo stage 2004				0.141
I, n (%)	11 (11.8%)	8 (14.5%)	3 (7.9%)	
II, n (%)	37 (39.8%)	25 (45.5%)	12 (31.6%)	
III, n (%)	45 (48.4%)	22 (40.0%)	23 (60.5%)	
Mayo stage 2012				0.331
I, n (%)	2(3.2%)	2(4.4%)	0(0)	
II, n (%)	9(14.2%)	8(17.8%)	1(5.6%)	
III, n (%)	26(41.3%)	19(42.2%)	7(38.9%)	
IV, n (%)	26(41.3%)	16(35.6%)	10(55.5%)	
Treatment strategy				0.213
Bortezomib based, n (%)	64 (68.8%)	39 (70.9%)	25 (65.8%)	
Melphalan based, n (%)	4 (4.3%)	2 (3.6%)	2 (5.3%)	
Immunomodulatory drugs based, n (%)	15 (16.1%)	9 (16.4%)	6 (15.8%)	
ASCT, n (%)	5 (5.4%)	4 (7.2%)	1 (2.6%)	
Others, n (%)	5 (5.4%)	1 (1.8%)	4 (10.5%)	
Hematologic response				0.205
Complete response, n (%)	36 (53.7%)	27 (61.4%)	9 (39.1%)	
Very good partial response, n (%)	14 (20.9%)	9 (20.5%)	5 (21.7%)	
Partial response, n (%)	13 (19.4%)	6 (13.6%)	7 (30.4%)	
No response, n (%)	4 (6.0%)	2 (4.5%)	2 (8.7%)	
MAGGIC risk score	19.0 ± 5.6	18.1 ± 5.3	20.3 ± 5.9	0.073

Biopsy and serum M protein results were obtained at initial diagnosis, other laboratory and demographic data were all tested within 1 week after diagnosis of amyloidosis for risk stratification and making treatment strategy

*ASCT* autologous stem cell transplantation, *BMI* body mass index, *dFLC* differential free light chains, *eGFR* estimated glomerular filtration rate, *MAGGIC* Meta-Analysis Global Group in Chronic Heart Failure, *NT-proBNP* N terminal pro B type natriuretic peptide, *NYHA* New York Heart Association

### Baseline echocardiographic features

Baseline conventional echocardiographic, tissue Doppler and LV speckle tracking analyses within 1 week after initial diagnosis of AL amyloidosis between survivors and non-survivors were displayed in Table 2. As required, all patients had a normal LVEF, but GLS significantly decreased (− 13.6 ± 4.7%). The LV wall thickness markedly increased (septal wall 15.6 ± 4.3 mm; posterior wall 14.8 ± 3.6 mm). LV size was generally normal. LV filling pressure increased as indicated by high E/e’ ratio of 18.7. According to the recommendations from the American Society of Echocardiography (ASE) [18], 13 (14.0%), 5 (5.4%) and 41 (44.1%) patients were classified as grade I, II and III LV diastolic dysfunction, respectively. Finally, only a minority of the patients experienced moderate (12.9%) or severe (7.5%) mitral regurgitation.

**Table 2:** Comparisons of baseline echocardiographic characteristics between survivors and non-survivors

Variables	Total (n = 93)	Survivors (n = 55)	Non-survivors (n = 38)	*P* value
LVEDD, mm	40.5 ± 5.6	41.4 ± 6.0	39.1 ± 5.0	0.059
LV IVS, mm	15.6 ± 4.3	15.3 ± 4.4	16.1 ± 4.1	0.365
LVPW, mm	14.8 ± 3.6	14.6 ± 3.5	14.9 ± 3.9	0.684
LVEDV/BSA, ml/m^2^	44.8 ± 15.9	48.4 ± 17.1	38.3 ± 14.0	0.049
LVESV/BSA, ml/m^2^	17.1 ± 9.0	18.3 ± 9.2	16.6 ± 8.6	0.428
LV mass/BSA, g/m^2^	160.3 ± 47.7	164.4 ± 51.9	154.8 ± 42.5	0.393
LVFS, %	34.0 ± 8.0	34.8 ± 8.4	32.8 ± 7.4	0.224
LVEF, %	59.7 ± 7.6	60.5 ± 8.0	58.6 ± 7.1	0.238
LV GLS, %	− 13.6 ± 4.7	− 14.8 ± 4.4	− 12.0 ± 4.7	0.004
Twist, °	12.7 ± 7.6	13.1 ± 8.1	12.2 ± 7.2	0.582
Dispersion, ms	56.4 ± 21.2	53.4 ± 18.6	61.6 ± 24.9	0.118
E wave, m/s	0.9 ± 0.3	0.8 ± 0.3	0.9 ± 0.3	0.433
A wave, m/s	0.6 ± 0.3	0.6 ± 0.3	0.5 ± 0.3	0.289
Tricuspid s’ wave, cm/s	10.8 ± 3.6	11.0 ± 3.3	10.7 ± 4.1	0.724
E/A	1.8 ± 0.9	1.6 ± 0.8	2.1 ± 1.0	0.017
E/e’ (lateral)	18.7 ± 8.9	15.7 ± 6.7	22.8 ± 14.7	0.009
TAPSE, mm	15.3 ± 4.4	16.1 ± 4.2	14.3 ± 4.5	0.054
TRV, m/s	2.2 ± 0.6	2.2 ± 0.7	2.1 ± 0.6	0.815
PASP, mmHg	30.5 ± 11.0	30.9 ± 11.4	29.9 ± 10.8	0.694
IVC, mm	17.2 ± 3.6	16.8 ± 3.7	17.8 ± 3.6	0.210
Pericardial effusion, n (%)	51 (54.8%)	27 (49.1%)	24 (63.2%)	0.180
Mitral regurgitation				0.769
None, n (%)	41 (44.1%)	25 (45.5%)	16 (42.1%)	
Mild, n (%)	33 (35.5%)	19 (34.5%)	16 (42.1%)	
Moderate, n (%)	12 (12.9%)	7 (12.7%)	5 (13.2%)	
Severe, n (%)	7 (7.5%)	4 (7.3%)	1 (2.6%)	
Diastolic dysfunction				0.067
None, n (%)	34 (36.6%)	24 (43.6%)	10 (26.3%)	
Grade I, n (%)	13 (14.0%)	10 (18.2%)	3 (7.9%)	
Grade II, n (%)	5 (5.4%)	2 (3.6%)	3 (7.9%)	
Grade III, n (%)	41 (44.1%)	19 (34.5%)	22 (57.9%)	
LA volume/BSA, ml/m^2^	32.1 ± 16.3	29.2 ± 8.4	36.5 ± 12.6	0.365
LA width, mm	43.2 ± 6.9	41.4 ± 5.5	45.9 ± 7.7	0.441

*BSA* body surface area, *GLS* global longitudinal strain, *IVC* inferior vena cava, *IVS* interventricular septum, *LA* left atrial, *LV* left ventricle, *LVEDD* left ventricular end-diastolic dimension, *LVEDV* left ventricular end-diastolic volume, *LVEF* left ventricular ejection fraction, *LVESV* left ventricular end-systolic volume, *LVFS* left ventricular fraction shortening, *LVPW* left ventricular posterior wall, *PASP* pulmonary artery systolic pressure, *TAPSE* tricuspid annular plane systolic excursion, *TRV* tricuspid regurgitation velocity

### LA mechanic indices

The average LA total, passive and active strains were 16.4 ± 11.0%, 8.4 ± 5.5% and 8.0 ± 7.2%, respectively. The interobserver variability of the speckle tracking assessment of LA mechanics based on the intraclass correlation coefficient (ICC) ranged from 0.81 to 0.94. The intraobserver variability based on the ICC ranged from 0.83 to 0.97.

**Fig. 2:**
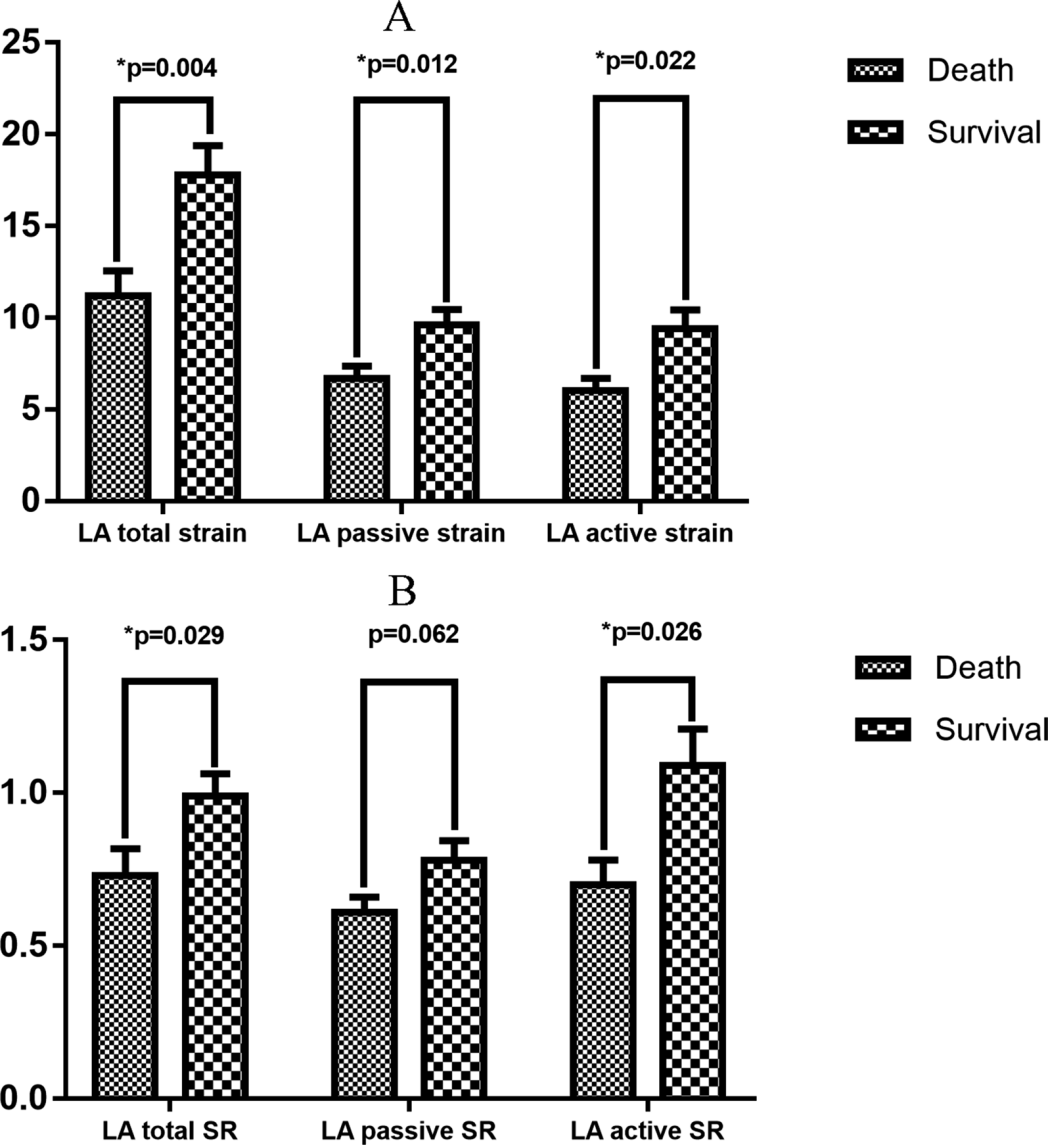
LA strain and strain rate in patients with different outcomes. **A** Comparison of LA strain; **B** comparison of LA strain rate. SR, strain rate

Compared with patients who survived until the last follow-up, LA total strain and strain rate, LA active strain and strain rate and LA passive strain were worse in patients who met the primary endpoint (Fig. 2). LA stiffness index was also significantly damaged in patients who died (3.31 ± 2.44 vs. 1.67 ± 1.49, *p* = 0.01).

### Prognostic utility of LA mechanics

In the univariate Cox regression analysis, as for the clinical indices, male gender, BMI, smoking, NYHA class 3 or 4 and NT-proBNP were associated with mortality (Additional file 1: Table S1). As for echocardiographic parameters, LV GLS, E/A, E/e’ and tricuspid annular plane systolic excursion (TAPSE) were associated with death. LA volume index (32.1 ± 16.3 ml/m^2^) and LA width (43.2 ± 6.9 mm) were increased but their association with mortality was not statistically significant on univariable analysis (Additional file 1: Table S2).

**Fig. 3:**
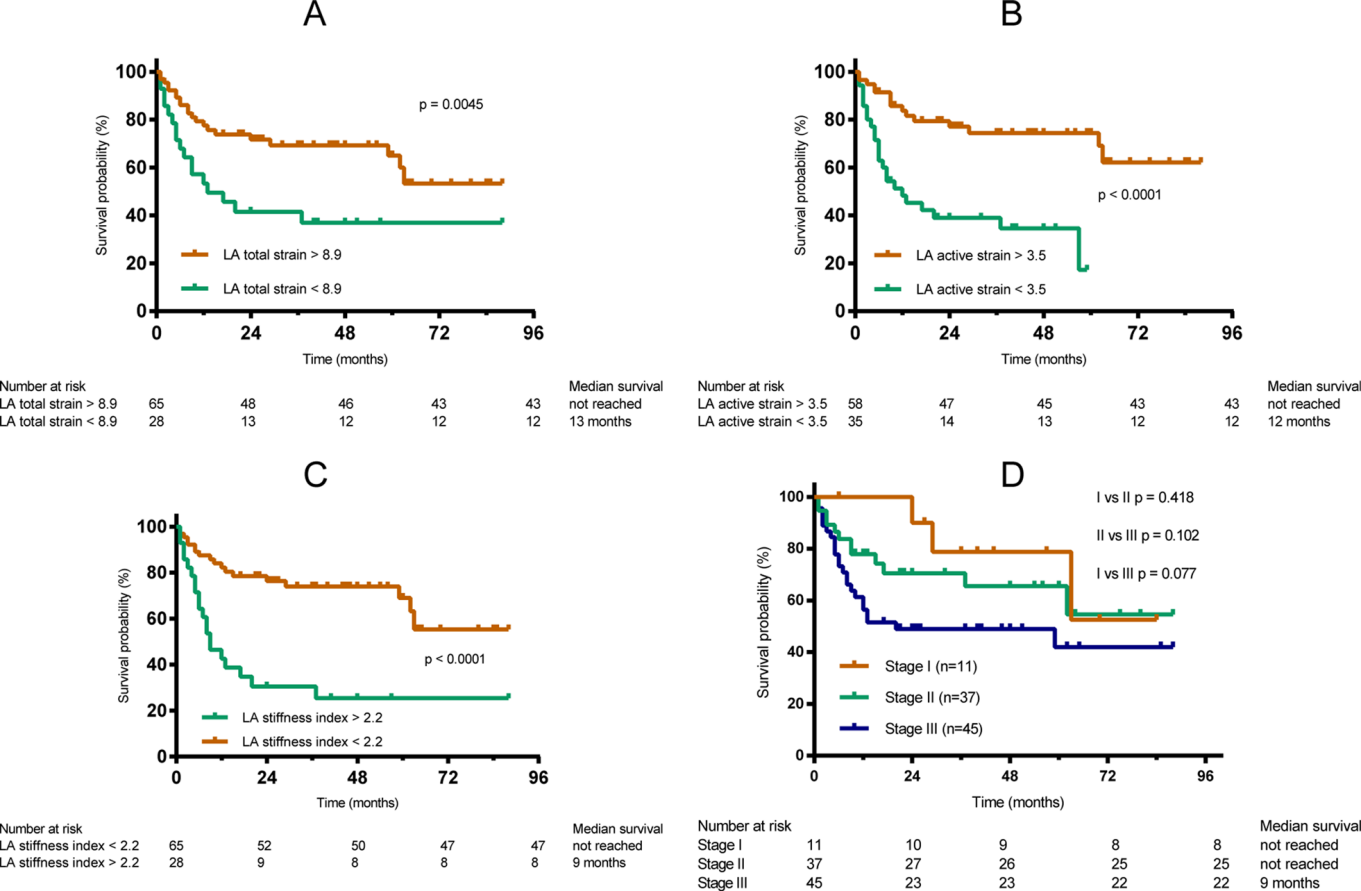
Kaplan–Meier curves for the probability of endpoint events. **A** In all patients, stratified by LA total strain; **B** in all patients, stratified by LA active strain; **C** in all patients, stratified by LA stiffness index; **D** in all patients, stratified by Mayo criteria 2004

In univariate Cox regression analysis for LA mechanics (Table 3), all indices except LA passive strain rate were correlated with survival. In the multivariate Cox regression analysis, after adjusting for other clinical and echocardiographic indices, LA mechanics regarding reservoir and booster pump functions (LA total strain, LA total strain rate, LA active strain, LA active strain rate) and LA stiffness index remained statistically significant. The ROC curve showed the optimal cut-off values for LA mechanics statistically significant in the multivariate Cox regression to predict the endpoint (Additional file 1: Table S3). Figure 3A–C illustrated overall survival according to the LA strain cut-off values in reservoir and contractile functional phases.

**Table 3:** Association of indices of LA mechanics with endpoint in patients with cardiac light-chain amyloidosis

Variables	Unadjusted		Adjusted (Model 1)		Adjusted (Model 2)	
	HR (95% CI)	*P* value	HR (95% CI)	*P* value	HR (95% CI)	*P* value
Total strain, %	0.944 (0.875–0.984)	0.005	0.920 (0.875–0.968)	0.001	0.923 (0.877–0.971)	0.002
Total strain rate, s^−1^	0.451 (0.223–0.911)	0.026	0.230 (0.091–0.578)	0.002	0.231 (0.089–0.603)	0.003
Passive strain, %	0.927 (0.867–0.991)	0.026	0.892 (0.820–0.971)	0.008	–	0.108
Passive strain rate, s^−1^	–	0.123	–	0.058	–	0.370
Active strain, %	0.928 (0.875–0.984)	0.013	0.879 (0.812–0.952)	0.001	0.883 (0.816–0.955)	0.002
Active strain rate, s^−1^	0.575 (0.348–0.951)	0.031	0.340 (0.165–0.700)	0.003	0.422 (0.195–0.914)	0.029
LA stiffness index	1.133 (1.044–1.229)	0.003	1.154 (1.064–1.252)	0.001	1.149 (1.059–1.247)	0.001

Model 1 adjusted for Meta-Analysis Global Group in Chronic Heart Failure risk score (including age, ejection fraction, creatinine, diabetes mellitus, chronic obstructive pulmonary disease, systolic blood pressure, body mass index, heart rate, current smoker, heart failure duration, New York Heart Association class, beta blocker use and angiotensin-converting enzyme inhibitor use) and Mayo stage 2004. Model 2 adjusted for model 1 variables + statistically significant echocardiographic indices in univariate Cox regression: GLS, E/A, E/e’ (lateral), and TAPSE

*CI* confidential interval, *HR* hazard ratio, *LA* left atrial

All strain values are presented as absolute values

### Incremental prognostic utility of LA mechanics

In our study, all patients could be stratified using the Mayo criteria 2004, while only 63 patients could be stratified with the Mayo criteria 2012 because examination of difference between the involved and uninvolved light-chain was available in our hospital since 2016. Mayo criteria 2004 roughly recognized the patients with a high prognostic risk (stage I versus II, *p* = 0.418; I versus III, *p* = 0.077) but was not statistically significant (Fig. 3D), so was Mayo criteria 2012 (stage I/II versus III, *p* = 0.19; I/II versus IV, *p* = 0.102). According to LA total strain, LA active strain and LA stiffness index, patients with Mayo criteria 2004 stage I/II or III were divided into 2 groups. In either stage I/II or III groups, the cut-off values of LA total strain < 8.88%, LA active strain < 3.48% or LA stiffness index ≥ 2.17 were all associated with a significantly higher risk of death (Additional file 1: Fig. S1).

As shown in Table 4, LA mechanics in reservoir and contractile phases outperformed the Mayo criteria 2004 and 2012 in their discrimination and goodness of fit. Both LA total strain and LA active strain had statistically significant absolute IDI and category-free NRI index statistic. LA active strain rate had the lowest BIC value.

**Table 4:** Incremental prognostic utility of LA mechanics compared with Mayo criteria 2004 and 2012

Variables	Mayo 2004	Mayo 2012	Reservoir function		Conduit function		Booster pump function		
			Total strain	Total strain rate	Passive strain	Passive strain rate	Active strain	Active strain rate	LA stiffness index
Discrimination (95% CI)									
C-statistic	0.62 (0.54–0.7)	0.66 (0.61–0.72)	0.68 (0.60–0.76)	0.67 (0.58–0.77)	0.65 (0.57–0.74)	0.65 (0.56–0.74)	0.68 (0.59–0.77)	0.67 (0.58–0.76)	0.69 (0.61–0.78)
			0.70 (0.64–0.76)	0.72 (0.67–0.78)	0.68 (0.62–0.73)	0.69 (0.63–0.76)	0.75 (0.71–0.81)	0.76 (0.70–0.81)	0.72 (0.66–0.77)
Absolute IDI	–	–	0.135 (0.040–0.280) *p* < 0.0001	0.08 (0.001–0.227) *p* = 0.03	0.061 (0.000–0.166) *p* = 0.05	0.027 (− 0.006–0.117) *p* = 0.159	0.116 (0.011–0.278) *p* < 0.0001	0.095 (0.001–0.236) *p* = 0.04	0.071 (0.004–0.175) *p* = 0.02
			0.105 (0.001–0.299) *p* = 0.036	0.134 (0.000–0.196) *p* = 0.05	0.077 (− 0.020–0.172) *p* = 0.144	0.021 (− 0.017–0.194) *p* = 0.219	0.063 (0.009–0.188) *p* = 0.02	0.046 (0.027–0.208) *p* = 0.018	0.062 (0.021–0.263) *p* = 0.009
Category-free NRI index	–	–	0.232 (0.046–0.494) *p* < 0.0001	0.273 (− 0.030–0.512) *p* = 0.06	0.189 (− 0.050–0.415) *p* = 0.179	0.122 (− 0.103–0.346) *p* = 0.209	0.241 (0.001–0.480) *p* = 0.04	0.208 (− 0.035–0.522) *p* = 0.09	0.364 (0.000–0.572) *p* = 0.05
			0.161 (0.034–0.387) *p* = 0.002	0.233 (− 0.069–0.580) *p* = 0.078	0.059 (− 0.251–0.401) *p* = 0.647	0.039 (− 0.276–0.360) *p* = 0.567	0.290 (0.006–0.413) *p* = 0.017	0.277 (0.002–0.567) *p* = 0.039	0.257 (0.026–0.434) *p* = 0.006
Goodness of fit									
LR test	–	–	*p* = 0.000124	*p* = 0.002329	*p* = 0.004477	*p* = 0.03902	*p* = 0.000408	*p* = 0.00152	*p* = 0.003526
			*p* = 0.02669	*p* = 0.002943	*p* = 0.08036	*p* = 0.05197	*p* = 0.005125	*p* = 0.002119	*p* = 0.01461
BIC	304.2324	134.9205	293.1057	277.5958	299.7637	282.6064	295.3481	276.8124	297.6163
			135.2916	112.6833	137.4963	118.4258	131.9916	112.026	133.1287

For each index, the upper line represents LA mechanics + Mayo criteria 2004 and the lower line represents LA mechanics + Mayo criteria 2012

*BIC* Bayes information criterion, *CI* confidential interval, *IDI* integrated discrimination index, *LA* left atrial, *NRI* net reclassification improvement

### Correlation between LA mechanic indices and other clinical and echocardiographic findings

As displayed in Supplementary data Additional file 1: Table S4, LA function was influenced by both LV systolic and diastolic function. NT-proBNP had strong correlations with LA total strain and LA passive strain. LV structure indices had weak correlations with LA function. Strong negative correlations between LA volume index and LA total strain, LA total strain rate, LA active strain and LA active strain rate were observed.

## Discussion

In this study, we evaluated the prognostic role of LA function in different functional phases in a cohort of cardiac light-chain amyloidosis patients with sinus rhythm and preserved EF. In our study, we found all components of LA strain except LA passive strain rate, as determined by 2D speckle tracking echocardiography, were worse in the group of patients who did not survive. LA strain and strain rate in the reservoir and contractile functional phases were independently predictive of death after adjusting for clinical and echocardiographic parameters and added prognostic value to the conventional Mayo criteria 2004 and 2012 in patients whose cardiac function was not yet at end stage.

In recent years, growing attention has been paid to the critical role of LA function on global cardiac performance. LA dysfunction is no longer treated as a bystander or merely the result of the deterioration of cardiac function [19]. The normal values, diagnostic and therapeutic utility of LA strain and strain rate in different diseases have gradually been elucidated, especially in heart failure with preserved ejection fraction (HFpEF) [9, 20–22]. Similar to HFpEF, CA patients commonly present with LV diastolic dysfunction, LA chamber enlargement and normal EF at a relatively early stage of the disease [5]. Furthermore, considering that amyloid infiltrates all chambers of the heart and not only LV deformation but also intrinsic LA amyloid infiltration damages LA function [7], LA might play a more independent and important role in CA than in HFpEF. Nochioka et al. investigated LA function in CA patients using 2D speckle tracking echocardiography [7]. The values of LA mechanics in our study population were comparable to those from the above study. Mohty et al. found a decrease in 3D peak atrial longitudinal strain was associated with worse two-year survival [23]. However, in CA patients, the prognostic utility of LA mechanics in different functional phases and whether they could add incremental prognostic value on Mayo criteria 2004 and 2012 were not investigated. Here, we performed a comprehensive analysis of the prognostic value of LA mechanics in cardiac light-chain amyloidosis patients with preserved EF and sinus rhythm.

First, LA reservoir and booster pump functions were more severely damaged than LA conduit function. LA reservoir and contractile indices were independently predictive of death, while LA passive strain rate was not associated with adverse outcomes. It is speculated that the preservation of LA conduit function allows the compensation of impaired LV filling in CA [24]. Second, Mohty et al. demonstrated that LA enlargement was an independent predictor for 5-year overall mortality in CA patients [5]. Zhao et al. also proved that LA enlargement was a significant predictor of all-cause mortality in cardiac AL amyloidosis and was strongly associated with the incidence of severe HF [8]. However, LA size does not equally represent its functional status [25]. It is difficult to determine whether an enlarged LA helps normalize cardiac function or, on the contrary, aggravate the process of decompensated heart failure. Thus, the direct measurement of LA function is critical. In our cohort, LA enlargement was observed but neither LA volume index nor LA width was associated with mortality. From our point of view, measurement of LA size is more convenient without offline analysis and has predictive value in the overall population having amyloidosis [5, 8]. But in patients at earlier disease stages, LA mechanics may be a more sensitive indicator of tissue infiltration and therefore a better marker of prognosis beyond and above LA size. Third, in clinical practice, the Mayo criteria 2004 and 2012 are widely recognized and used for risk stratification. They are readily available and reproducible for identifying patients at high risk and guiding therapeutic decisions [26, 27]. In our study, patients with LVEF < 50% and atrial fibrillation were excluded, leading to a less advanced global disease stage of the study population. In this population, patients with a more advanced disease stage based on the Mayo criteria 2004 and 2012 exhibited a tendency towards worse outcomes, but this trend was not statistically significant. LA reservoir and booster pump functional indices helped precisely identify patients at high risk and added incremental prognostic value.

Our study had several limitations. First, this was a retrospective study carried out in a single center with a limited sample size. However, we managed to achieve over 15-fold more samples compared to parameters in the multivariate Cox regression for this rare disease. Second, patients with atrial arrhythmias and impaired ejection fraction were excluded from our study. The prognostic value of LA size has been verified in amyloidosis, we targeted a specific subgroup of patients to test its predictive value in less advanced disease and demonstrated LA mechanics were more sensitive than LA size in these patients. The findings of our study should be extended to other population of CA patients with caution. Third, patients with mitral regurgitation, another contributing pathology that may affect LA mechanics, were included in our cohort [28]. However, only approximately 20% of patients experienced moderate or severe mitral regurgitation in the present study, and was not statistically significant in univariate analyses. Fourth, the acquisition of LA strain is not well defined. We used the method consistent with previous studies [7, 20–22]. Finally, this study did not contain echocardiographic follow-up results, which could be used to evaluate the changes in LA mechanics during treatment and to further assess their prognostic utility; thus, such results are warranted in future prospective studies.

## Conclusions

In patients with cardiac light-chain amyloidosis, preserved EF and a normal sinus rhythm, LA reservoir and booster pump functional indices are independently associated with overall survival. They could add incremental prognostic utility to the model used for risk stratification in clinical practice, the Mayo criteria 2004 and 2012. Considering these findings, emphasizing the monitoring of LA function may be beneficial for the prognosis prediction of cardiac light-chain amyloidosis.

## Supplementary Information

**Additional file 1.** **Table S1**. Univariate COX regression of demographic and clinical characteristics in patients with cardiac light-chain amyloidosis; **Table S2**. Univariate COX regression of baseline echocardiographic characteristics in patients with cardiac light-chain amyloidosis; **Table S3**. Cut-off values for LA mechanics to predict overall survival; **Table S4**. Correlations between LA mechanics and other clinical and echocardiographic findings; **Figure S1**. Kaplan-Meier curves for the probability of endpoint events. (A) in patients with Mayo criteria stages 1 and 2, stratified by LA active strain; (B) in patients with Mayo criteria stage 3, stratified by LA active strain; (C) in patients with Mayo criteria stages 1 and 2, stratified by LA stiffness index; (D) in patients with Mayo criteria stage 3, stratified by LA stiffness index; (E) in patients with Mayo criteria stages 1 and 2, stratified by LA total strain; (F) in patients with Mayo criteria stage 3, stratified by LA total strain.

## Data Availability

The datasets used and/or analyzed during the current study are available from the corresponding author on reasonable request.

## Abbreviations

AL: Light-chain amyloidosis
ATTR: Transthyretin amyloidosis
BIC: Bayes information criteria
CA: Cardiac amyloidosis
EF: Ejection fraction
EMB: Endomyocardial biopsy
GLS: Global longitudinal strain
HFpEF: Heart failure with preserved ejection fraction
ICC: Intraclass correlation coefficient
IDI: Integrated discrimination improvement
LA: Left atrial
LV: Left ventricular
NRI: Net reclassification improvement
OS: Overall survival
TAPSE: Tricuspid annular plane systolic excursion
