# Supplementary material for: Prognostic value of left atrial mechanics in cardiac light-chain amyloidosis with preserved ejection fraction: a cohort study
Source: BMC Cardiovasc Disord. 2022 Apr 15;22:175. doi: 10.1186/s12872-022-02589-7 (PMC9013068; doi:10.1186/s12872-022-02589-7)

**Additional file 1**

**Table S1. Univariate COX regression of demographic and clinical characteristics in patients with cardiac light-chain amyloidosis**

| Variables | Total (n = 93) | HR (95% CI) | P value |
| --- | --- | --- | --- |
| Age, years | 58.6 ± 9.4 | 1.014 (0.978-1.050) | 0.453 |
| Male sex, n (%) | 50 (53.8%) | 1.988 (1.030-3.835) | 0.040 |
| Systolic blood pressure, mmHg | 105.3 ± 16.2 | 0.992 (0.972-1.013) | 0.446 |
| Diastolic blood pressure, mmHg | 68.5 ± 10.4 | 0.995 (0.963-1.027) | 0.736 |
| Heart rate, bpm | 82.0 ± 13.6 | 1.015 (0.992-1.039) | 0.193 |
| BMI, kg/m^2^ | 22.3 ± 3.0 | 1.122 (1.009-1.248) | 0.034 |
| Smoking, n (%) | 34 (36.6%) | 2.318 (1.178-4.558) | 0.015 |
| eGFR, ml/min/1.73㎡ | 97.2 ± 32.6 | 0.997 (0.987-1.007) | 0.523 |
| NYHA class 3 or 4, n (%) | 36 (38.7%) | 2.622 (1.315-5.229) | 0.006 |
| NT-proBNP, median [IQR]* | 2514 [818-7545] | 1.339 (1.124-1.741) | 0.003 |
| Intact M protein, n (%) | 37 (39.8%) | 1.032 (0.941-1.132) | 0.508 |
| Intact M concentration, g/L, median [IQR] | 4.7 [2.0-9.1] | 0.859 (0425-1.738) | 0.673 |
| Bone marrow plasma cells, %, median [IQR] | 4.0 [2.3-10.0] | 0.987 (0.924-1.055) | 0.706 |
| dFLC, mg/L, median [IQR]* | 216.2 [90-387] | 1.155 (0.832-1.603) | 0.388 |
| Mayo stage 2004  Comparison with I, n (%)  II, n (%)  III, n (%) | 11 (11.8%)  37 (39.8%)  45 (48.4%) | 1.00 (ref)  1.800 (0.509-6.366)  2.824 (0.841-9.487) | ...  0.362  0.093 |
| Mayo stage 2012  Comparison with I/II, n (%)  III, n (%)  IV, n (%) | 11(17.4%)  26(41.3%)  26(41.3%) | 1.00 (ref)  4.092(0.498-33.589)  5.596(0.712-43.984) | ...  0.190  0.102 |
| Involved light chain type  λ, n (%)  κ, n (%) | 78 (83.9%)  15 (16.1%) | 1.00 (ref)  0.620 (0.238-1.616) | ...  0.328 |
| Other organ involvement  Kidney, n (%)  Liver, n (%)  PNS, n (%)  Gastrointestinal tract, n (%)  >2 organs, n (%) | 48 (51.6%)  22 (23.7%)  19 (20.4%)  8 (8.6%)  20 (21.4%) | 0.661 (0.333-1.309)  1.616 (0.767-3.405)  1.042 (0.451-2.408)  0.197 (0.027-1.446)  0.950 (0.390-2.318) | 0.235  0.207  0.924  0.110  0.911 |
| Treatment strategy  Comparison with bortezomib based, n (%)  Melphalan based, n (%)  Immunomodulatory drugs based, n (%)  ASCT, n (%)  Others, n (%) | 64 (68.8%)  4 (4.3%)  15 (16.1%)  5 (5.4%)  5 (5.4%) | 1.00 (ref)  0.728 (0.097-5.433)  1.233 (0.483-3.099)  1.429 (0.333-6.138)  5.365 (1.760-16.36) | ...  0.757  0.671  0.631  0.003 |
| Cardiac medication  Beta blocker, n (%)  Ca blocker, n (%)  RAS-I, n (%)  Diuretics, n (%)  Statins, n (%) | 14 (15.1%)  5 (5.4%)  17 (18.3%)  51 (54.8%)  11 (11.8%) | 0.917 (0.358-2.350)  0.340 (0.046-2.483)  1.268 (0.600-2.681)  2.104 (0.997-4.361)  1.472 (0.613-3.535) | 0.857  0.287  0.534  0.104  0.387 |
| Hematologic response  Comparison with complete response  Very good partial response  Partial response  No response | 36 (53.7%)  14 (20.9%)  13 (19.4%)  4 (6.0%) | 1.00 (ref)  1.836 (0.625-5.394)  2.385 (0.905-6.287)  5.305 (1.102-25.529) | ...  0.269  0.079  0.037 |
| MAGGIC risk score | 19.0 ± 5.6 | 1.076 (1.009-1.148) | 0.025 |

ASCT, autologous stem cell transplantation; BMI, body mass index; CI, confidential interval; dFLC, differential free light chains; eGFR, estimated glomerular filtration rate; HR, hazard ratio; MAGGIC, Meta-Analysis Global Group in Chronic Heart Failure; NT-proBNP, N terminal pro B type natriuretic peptide; NYHA, New York Heart Association; PNS, peripheral nervous system; RAS-I, rennin angiotensin system inhibitor. *HR is per 1-SD increase.

**Table S2. Univariate COX regression of baseline echocardiographic characteristics in patients with cardiac light-chain amyloidosis**

| Variables | Total (n = 93) | HR (95% CI) | P value |
| --- | --- | --- | --- |
| LVEDD, mm | 40.5 ± 5.6 | 0.953 (0.905-1.004) | 0.073 |
| LV IVS, mm | 15.6 ± 4.3 | 1.046 (0.972-1.126) | 0.232 |
| LVPW, mm | 14.8 ± 3.6 | 1.032 (0.946-1.126) | 0.481 |
| LVEDV/BSA, ml/m^2^ | 44.8 ± 15.9 | 0.987 (0.974-1.001) | 0.064 |
| LVESV/BSA, ml/m^2^ | 17.1 ± 9.0 | 0.994 (0.970-1.018) | 0.627 |
| LV mass/BSA, g/m^2^ | 160.3 ± 47.7 | 0.998 (0.991-1.005) | 0.540 |
| LVFS, % | 34.0 ± 8.0 | 0.967 (0.928-1.007) | 0.109 |
| LVEF, % | 59.7 ± 7.6 | 0.973 (0.931-1.017) | 0.225 |
| LV GLS, % | -13.6 ± 4.7 | 1.143 (1.054-1.239) | 0.001 |
| Twist, ° | 12.7 ± 7.6 | 0.977 (0.930-1.027) | 0.365 |
| Dispersion, ms | 56.4 ± 21.2 | 1.011 (0.995-1.028) | 0.180 |
| E wave, m/s | 0.9 ± 0.3 | 1.649 (0.538-5.055) | 0.382 |
| A wave, m/s | 0.6 ± 0.3 | 0.366 (0.094-1.430) | 0.148 |
| Tricuspid s’ wave, cm/s* | 10.8 ± 3.6 | 0.910 (0.655-1.263) | 0.572 |
| E/A | 1.8 ± 0.9 | 1.592 (1.137-2.229) | 0.007 |
| E/e’ (lateral) | 18.7 ± 8.9 | 1.028 (1.009-1.046) | 0.003 |
| TAPSE, mm | 15.3 ± 4.4 | 0.917 (0.847-0.993) | 0.033 |
| TRV, m/s | 2.2 ± 0.6 | 0.957 (0.589-1.554) | 0.859 |
| PASP, mmHg | 30.5 ± 11.0 | 0.995 (0.967-1.024) | 0.733 |
| IVC, mm | 17.2 ± 3.6 | 1.079 (0.983-1.184) | 0.108 |
| Pericardial effusion, n (%) | 51 (54.8%) | 1.618 (0.838-3.124) | 0.152 |
| Mitral regurgitation  Comparison with none, n (%)  Mild, n (%)  Moderate, n (%)  Severe, n (%) | 41 (44.1%)  33 (35.5%)  12 (12.9%)  7 (7.5%) | 1.00 (ref)  1.010 (0.509-2.004)  0.805 (0.296-2.186)  1.315 (0.484-3.571) | ...  0.978  0.670  0.591 |
| Diastolic dysfunction  Comparison with none, n (%)  Grade I, n (%)  Grade II, n (%)  Grade III, n (%) | 34 (36.6%)  13 (14.0%)  5 (5.4%)  41 (44.1%) | 1.00 (ref)  1.077 (0.951-1.156)  0.941 (0.853-1.034)  1.441 (0.947-1.953) | ...  0.141  0.412  0.129 |
| LA volume/BSA, ml/m^2^ | 32.1 ± 16.3 | 1.009 (0.995-1.022) | 0.212 |
| LA width, mm | 43.2 ± 6.9 | 1.041 (0.967-1.127) | 0.241 |

BSA, body surface area; CI, confidential interval; GLS, global longitudinal strain; HR, hazard ratio; IVC, inferior vena cava; IVS, interventricular septum; LA, left atrial; LV, left ventricle; LVEDD, left ventricular end-diastolic dimension; LVEDV, left ventricular end-diastolic volume; LVEF, left ventricular ejection fraction; LVESV, left ventricular end-systolic volume; LVFS, left ventricular fraction shortening; LVPW, left ventricular posterior wall; PASP, pulmonary artery systolic pressure; TAPSE, tricuspid annular plane systolic excursion; TRV, tricuspid regurgitaition velocity; *HR is per 1-SD increase.

**Table S3. Cut-off values for LA mechanics to predict overall survival**

|  | Total strain | Total strain rate | Active strain | Active strain rate | LA stiffness index |
| --- | --- | --- | --- | --- | --- |
| Cut-off values | 8.88% | 0.661 | 3.48% | 0.535 | 2.17 |
| AUC | 0.682 | 0.687 | 0.685 | 0.676 | 0.685 |
| Specificity | 0.513 | 0.676 | 0.615 | 0.676 | 0.749 |
| Sensitivity | 0.778 | 0.660 | 0.778 | 0.680 | 0.513 |
| Hazard ratio (95% CI) | 2.41 (1.16-5.02) | 3.02 (1.55-5.89) | 3.67 (1.83-7.37) | 3.44 (1.76-6.70) | 3.66 (1.72-7.76) |
| P value | 0.0045 | 0.0008 | < 0.0001 | 0.0003 | < 0.0001 |

AUC, area under the curve; CI, confidential interval.

**Table S4. Correlations between LA mechanics and other clinical and echocardiographic findings**

| Variables | Pearson’s correlation (P value) | | | | | | |
| --- | --- | --- | --- | --- | --- | --- | --- |
|  | Reservoir function | | Conduit function | | Active function | |  |
|  | Total strain, % | Total strain rate, s^-1^ | Passive strain, % | Passive strain rate, s^-1^ | Active strain, % | Active strain rate, s^-1^ | LA stiffness index |
| Age | -0.033 (p = 0.755) | -0.065 (p = 0.55) | -0.044 (p = 0.677) | -0.023 (p = 0.834) | 0.042 (p = 0.693) | 0.016 (p = 0.883) | 0.017 (p = 0.870) |
| Heart rate | -0.254 (p = 0.014) | -0.156 (p = 0.148) | -0.239 (p = 0.021) | 0.022 (p = 0.839) | -0.216 (p = 0.037) | -0.2 (p = 0.064) | 0.097 (p = 0.359) |
| SBP | 0.176 (p = 0.115) | 0.062 (p = 0.595) | 0.111 (p = 0.324) | 0.064 (p = 0.583) | 0.183 (p = 0.102) | 0.126 (p = 0.282) | -0.032 (p = 0.781) |
| NT-proBNP | -0.41 (p < 0.0001) | -0.368 (p = 0.001) | -0.378 (p< 0.0001) | -0.271 (p = 0.013) | -0.344 (p = 0.001) | -0.339 (p = 0.002) | 0.172 (p = 0.109) |
| LV structure | | | | | | | |
| LVEDV/BSA | 0.145 (p = 0.2) | 0.214 (p = 0.065) | 0.052 (p = 0.647) | 0.005 (p = 0.936) | 0.194 (p = 0.085) | 0.2 (p = 0.086) | -0.128 (p = 0.229) |
| LV mass index/BSA | 0.022 (p = 0.849) | -0.009 (p = 0.939) | -0.016 (p = 0.891) | -0.143 (p = 0.221) | 0.079 (p = 0.5) | 0.012 (p = 0.919) | 0.127 (p = 0.280) |
| LV function | | | | | | | |
| LV GLS | 0.630 (p < 0.0001) | 0.514 (p < 0.0001) | 0.514 (p < 0.0001) | 0.37 (p < 0.0001) | 0.508 (p < 0.0001) | 0.514 (p < 0.0001) | -0.468 (p < 0.0001) |
| LVEF | 0.377 (p < 0.0001) | 0.339 (p = 0.001) | 0.26 (p = 0.12) | 0.106 (p = 0.332) | 0.35 (p = 0.001) | 0.365 (p = 0.001) | -0.174 (p = 0.099) |
| E wave | -0.336 (p = 0.001) | -0.321 (p = 0.003) | -0.228 (p = 0.029) | -0.091 (p = 0.406) | -0.371 (p< 0.0001) | -0.428 (p< 0.0001) | 0.465 (p < 0.0001) |
| A wave | 0.365 (p < 0.0001) | 0.38 (p < 0.0001) | 0.233 (p = 0.025) | 0.048 (p = 0.662) | 0.407 (p < 0.0001) | 0.333 (p = 0.002) | -0.178 (p = 0.089) |
| E/A | -0.572 (p< 0.0001) | -0.575 (p< 0.0001) | -0.374 (p< 0.0001) | -0.171 (p = 0.115) | -0.622 (p< 0.0001) | -0.596 (p< 0.0001) | 0.566 (p < 0.0001) |
| E/e’ | -0.453 (p< 0.0001) | -0.351 (p = 0.001) | -0.458 (p< 0.0001) | -0.412 (p< 0.0001) | -0.325 (p = 0.002) | -0.312 (p = 0.003) | 0.790 (p < 0.0001) |
| LA structure | | | | | | | |
| LA volume index | -0.426 (p< 0.0001) | -0.51 (p< 0.0001) | -0.331 (p = 0.001) | -0.225 (p = 0.036) | -0.461 (p< 0.0001) | -0.481 (p< 0.0001) | 0.231 (p = 0.027) |

BSA, body surface area; GLS, global longitudinal strain; LA, left atrial; LV, left ventricle; LVEDV, left ventricular end-diastolic volume; LVEF, left ventricular ejection fraction; NT-proBNP, N terminal pro B type natriuretic peptide; SBP, systolic blood pressure.

**Figure S1. Kaplan-Meier curves for the probability of endpoint events.** (A) in patients with Mayo criteria stages 1 and 2, stratified by LA active strain; (B) in patients with Mayo criteria stage 3, stratified by LA active strain; (C) in patients with Mayo criteria stages 1 and 2, stratified by LA stiffness index; (D) in patients with Mayo criteria stage 3, stratified by LA stiffness index; (E) in patients with Mayo criteria stages 1 and 2, stratified by LA total strain; (F) in patients with Mayo criteria stage 3, stratified by LA total strain.


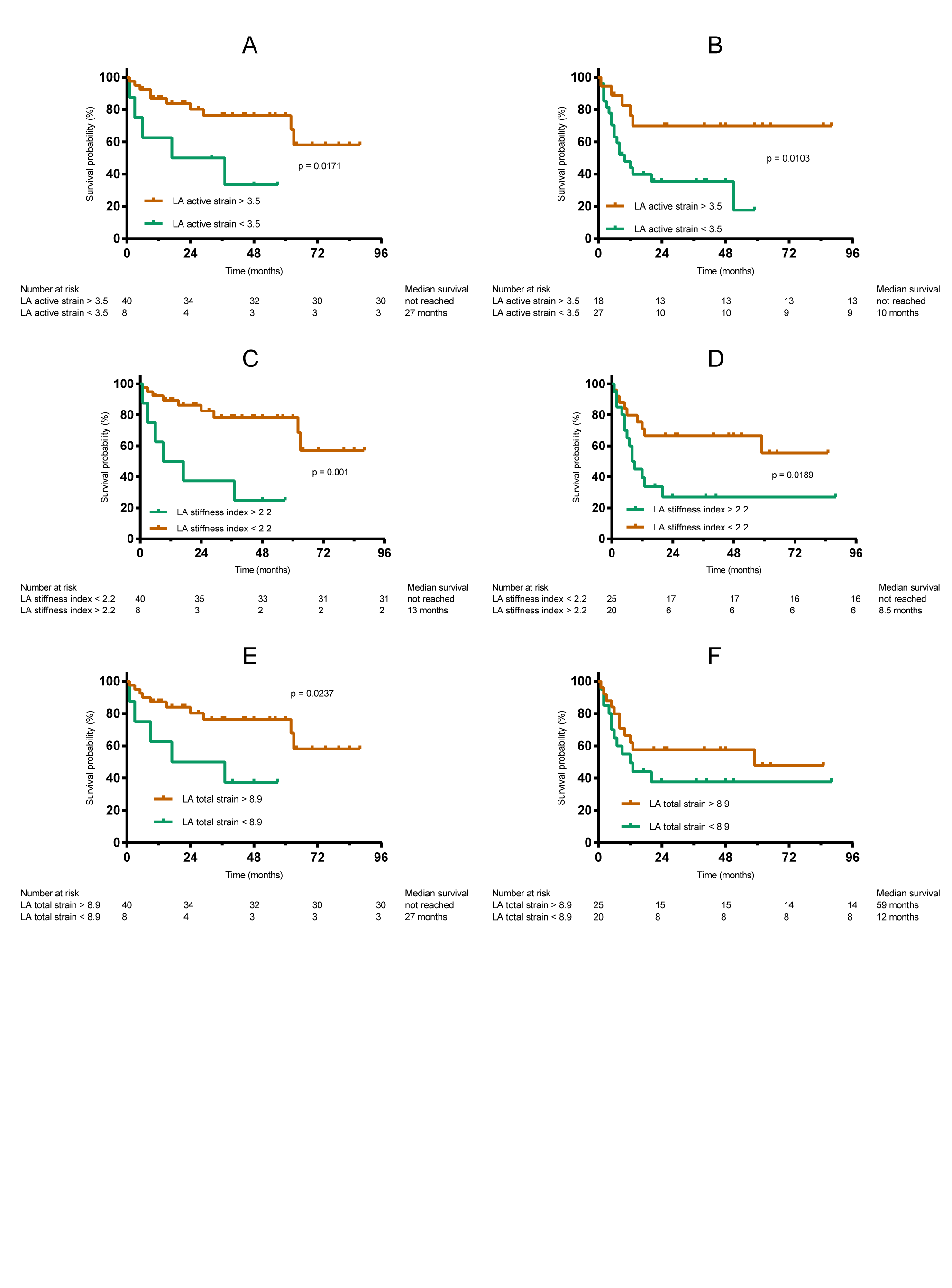

Supplement: Supplementary file 1 — Additional file 1. Table S1. Univariate COX regression of demographic and clinical characteristics in patients with cardiac light-chain amyloidosis; Table S2. Univariate COX regression of baseline echocardiographic characteristics in patients with cardiac light-chain amyloidosis; Table S3. Cut-off values for LA mechanics to predict overall survival; Table S4. Correlations between LA mechanics and other clinical and echocardiographic findings; Figure S1. Kaplan-Meier curves for the probability of endpoint events. (A) in patients with Mayo criteria stages 1 and 2, stratified by LA active strain; (B) in patients with Mayo criteria stage 3, stratified by LA active strain; (C) in patients with Mayo criteria stages 1 and 2, stratified by LA stiffness index; (D) in patients with Mayo criteria stage 3, stratified by LA stiffness index; (E) in patients with Mayo criteria stages 1 and 2, stratified by LA total strain; (F) in patients with Mayo criteria stage 3, stratified by LA total strain. [file 12872_2022_2589_MOESM1_ESM.docx]
